# Supplementary material for: Surgical and Medical Aspects of the Initial Treatment of Biliary Atresia: Position Paper
Source: J Clin Med. 2022 Nov 7;11(21):6601. doi: 10.3390/jcm11216601 (PMC9656543; doi:10.3390/jcm11216601)
Supplement: Supplementary file 1 [file jcm-11-06601-s001.zip › jcm-1978360-supplementary.pdf]

1. Technique general

- ◯ Open surgery
- ◯ MIS - Cholangiography before Kasai
- ◯ MIS complete

2. Cholangiography

- ◯ Obligatory
- ◯ When ERCP not available or inconclusive
- ◯ Only when macroscopic inspection of the hepatoduodenal ligament is inconclusive
- ◯ Never

3. Do you perform indocyanine green cholangiogram?

- ◯ Yes
- ◯ No

4. Role of indocyanine green cholangiogram?

[ ]

5. Full assessment for associated pathology (BASM, malrotation, etc.)

- ◯ Yes
- ◯ No

6. Survey answered by Kasai surgeon

- ◯ Yes
- ◯ No

Open procedure

7. Mobilisation of the liver

- ◯ Total
- ◯ Partial
- ◯ No

8. Using magnification during the dissection of the porta hepatis

- ◯ Yes
- ◯ No

9. Using knife during the dissection of the porta hepatis

- ◯ Yes
- ◯ No

10. Using bipolar coagulation during the dissection of the porta hepatis

- ◯ Yes
- ◯ No

11. Exposure of the Recessus Rex

- ◯ Yes
- ◯ No
- ◯ Optional

12. Do you perform extended dissection (up to first branching of the portal vein)

- ◯ Yes
- ◯ No
- ◯ Optional

13. Role of indocyanine green to evaluate dissection of liver surface

[ ]

14. Other techniques during dissection of liver surface (please specify)

[ ]

15. Roux-n-Y lenghth

- ◯ < 25cm
- ◯ 25 - 50cm
- ◯ > 50cm

16. Construction of valve

- ◯ Yes
- ◯ No

17. If yes, that type of valve

[ ]

18. Roux-en-Y technique

- ◯ Side-to-Side
- ◯ End-to-Side

19. Roux-en-Y anastomosis

- ◯ Stapled
- ◯ Hand-sewn

20. If hand-sewn

- ◯ Running sutures
- ◯ Interrupted sutures

21. Position of the Roux-n-Y-loop with regard to later liver transplantation

- ◯ retrocolic
- ◯ antecolic

22. Kasai anastomosis

- ◯ Interrupted sutures
- ◯ Running sutures
- ◯ Both (please specify)

23. Kasai anastomosis technique

- ◯ End-to-Side
- ◯ End-to-End
- ◯ Both (please specify)

24. Size of sutures

- ◯ 5/0
- ◯ 6/0
- ◯ 7/0
- ◯ Other (please specify)

25. Placement of drain

- ◯ Yes
- ◯ No
- ◯ Optional

26. Liver biopsy (simultaneously with the Kasai)

- ◯ Wedge
- ◯ Needle
- ◯ Both
- ◯ No biopsy

27. Pathological assessment of excised portal plate

- ◯ Yes
- ◯ No

28. Bio bank

- ◯ Yes
- ◯ No

29. Duration of open Kasai

- ◯ < 120 min
- ◯ 120 – 240 min
- ◯ > 240 min

30. Duration of MIS Kasai

- ◯ < 120 min
- ◯ 120 – 240 min
- ◯ > 240 min

31. Perioperative prophylaxis - Substance:

[ ]

32. Duration of perioperative prophylaxis

- ◯ 1 - 3 days
- ◯ 1 week
- ◯ 2 weeks
- ◯ > 2 weeks

33. Postoperative nasogastric tube

- ◯ Yes
- ◯ No

34. Start feeding

- ◯ Next day
- ◯ After bowel movement
- ◯ Other indicator (please specify)

[ ]

35. Tube feeding

- ◯ Yes
- ◯ No
- ◯ Optional

36. Type of feeding

- ◯ Breast milk
- ◯ High MCT-containing formula
- ◯ Other (please specify)

37. Corticosteroids

- ◯ Yes
- ◯ No

38. If yes, please specify substance

39. Dosage tapered, please specify

- ◯ 1 week
- ◯ 2 weeks
- ◯ 2 - 4 weeks
- ◯ > 4 weeks

40. Immunoglobuline

- ◯ Yes
- ◯ No

41. Dosage and Duration of Immunoglobuline

42. Farnesoid X receptor (FXR) agonist

- ◯ Yes
- ◯ No

43. Dosage and Duration of Farnesoid X receptor (FXR) agonist

44. Ileal bile acid transporter/ apical sodium–bile acid transporter (IBAT,ASBT) inhibitors

- ◯ Yes
- ◯ No

45. Dosage and Duration of IBAT / ASBT inhibitors

46. Others/experimental therapy of interest (please specify)

47. Ursodeoxycholic acid

- ○ Yes
- ○ No

48. Ursodeoxycholic acid treatment duration

49. Antibiotic cholangitis prophylaxis

- ○ Yes
- ○ No

50. Type and duration of antibiotic treatment

51. Routine follow-up schedule

- ○ Yes
- ○ No

52. Follow-up schedule - Duration

53. Who is involved in the follow-up

- ☐ Pediatric gastroenterology
- ☐ Pediatric surgery
- ☐ Other (please specify)

54. For patients, who survive with native liver, is there a transition program to adult hepatology established

- ○ Yes
- ○ No

55. Is the Kasai surgeon involved in liver transplantation

- ○ Yes
- ○ No
